# Adherence to micronutrient powder for home fortification of foods among infants and toddlers in rural China: a structural equation modeling approach

**DOI:** 10.1186/s12889-022-14731-3

**Published:** 2022-12-02

**Authors:** Chang Sun, Ruixue Ye, Muizz Akhtar, Sarah-Eve Dill, Ping Yuan, Huan Zhou, Scott Rozelle

**Affiliations:** 1 Department of Epidemiology and Health Statistics, West China School of Public Health and West China Fourth Hospital, Sichuan University, 610041 Chengdu, Sichuan China; 2 Department of Health Behavior and Social Medicine, West China School of Public Health, West China Fourth Hospital, Sichuan University, 610041 Chengdu, Sichuan China; 3 Stanford Center on China’s Economy and Institutions, Stanford University, Stanford, CA USA

**Keywords:** China, Micronutrient Powder, Ying Yang Bao, Infants and toddlers, Integrated behavioral model, Structural equation modeling, Adherence

## Abstract

**Background:**

The WHO recommends daily use of micronutrient powder for infants and toddlers at risk of micronutrient deficiencies in low-and-middle-income countries. China has established a micronutrient powder distribution program in many rural townships and villages, yet adherence to micronutrient powder remains suboptimal; a little is known about the behavioral inputs that may influence adherence. This study examines direct and indirect behavioral inputs in micronutrient powder adherence among caregivers in rural western China following the Integrated Behavioral Model (IBM) framework.

**Methods:**

Cross-sectional data were collected from April to May 2019 among 958 caregivers of children aged 6 to 24 months in six counties. Data were collected on micronutrient powder adherence behavior, direct behavioral inputs (knowledge and skills, intention, salience, environmental constraints, and habits), and indirect behavioral inputs (attitudes, perceived social norms, and personal agency). Structural equation modeling (SEM) adjusted for sociodemographic covariates was used to evaluate the IBM framework.

**Results:**

Mean micronutrient powder adherence in the previous seven days was 53.02%, and only 22.86% of caregivers consistently fed micronutrient powder from the start of micronutrient powder distribution at six months of age. The SEM model revealed small- to medium-sized effects of salience (β = 0.440, P < 0.001), intention (β = 0.374, *P* < 0.001), knowledge and skills (β = 0.214, *P* < 0.001), personal agency (st. effect = 0.172, *P* < 0.001), environmental constraints (β=-0.142, *P* < 0.001), and caregiver generation (β = 0.119, *P* < 0.05) on micronutrient powder adherence. Overall, 54.7% of the variance in micronutrient powder adherence was explained by the IBM framework. Salience had the largest impact on micronutrient powder adherence (Cohen’s *f* ^2^ = 0.227). Compared to parent caregivers, grandparents had a higher degree of micronutrient powder adherence on average (*P* < 0.001), and behavioral inputs were consistent among both parent and grandparent caregivers.

**Conclusion:**

There is a need to improve micronutrient powder adherence among rural caregivers. The IBM framework showed a high degree of explanatory power in predicting micronutrient powder adherence behavior. The findings suggest that increased reminders from doctors regarding micronutrient powder and coaching to improve personal agency in micronutrient powder feeding may increase adherence.

**Supplementary Information:**

The online version contains supplementary material available at 10.1186/s12889-022-14731-3.

## Background

Micronutrient deficiencies due to inadequate food consumption and a lack of dietary diversity are a significant concern in preschool-aged children, resulting in wide-ranging adverse health effects, especially in low- and middle-income countries (LMICs) [1, 2]. To reduce the prevalence of micronutrient deficiencies in LMICs, the WHO recommends the daily administration of micronutrient powders (MNP) to at-risk children. MNP are single dose sachet of dry powder containing iron and other micronutrients that can be sprinkled on semisolid food of children [3]. Over the last decade, MNP distribution programs have been shown to effectively reduce iron deficiency anemia among young children in LMICs by more than 50% when caregivers adhere to regular feeding practices [3–6]. However, there are concerns that low rates of MNP adherence may dilute would-be program benefits and limit the overall effectiveness of MNP distribution [7–9].

Like other LMICs, China implemented a free MNP distribution program for children in 2012. Locally marketed at Ying Yang Bao, the MNP contains protein, calcium, iron, zinc, vitamin A, vitamin D, vitamin B1, vitamin B2, vitamin B12, and folic acid. The program was initially implemented in 100 nationally designated poverty counties and has since expanded to 715 counties in 21 provinces across China. Chinese MNP feeding guidelines stipulate that caregivers should administer four to seven MNP sachets to their infants every week [10], and previous studies have found that regular adherence to these guidelines can effectively reduce anemia among young children [11].

However, there is evidence that caregiver adherence to MNP feeding guidelines is low, especially in western China where childhood iron deficiency anemia remains prevalent. Across western provinces, studies have shown that the rate of anemia among children under age two is over 50% [12], yet only half of caregivers in western China report feeding their children MNP at least four times per week [11, 13, 14]. With such low rates of adherence to MNP feeding guidelines, it is likely that the MNP program is not achieving its desired health impacts on infants and toddlers in western China.

To address these low rates of MNP adherence in western China, a comprehensive understanding of adherence to the MNP program is urgently needed. Previous studies conducted in China have found significant relationships between MNP adherence and caregiver characteristics such as ethnicity, educational attainment, as well as infant age and infant acceptance of the taste of MNP [11, 13–15]. However, less is known about the behavioral inputs that may influence MNP adherence. The international literature points to a combination of behavioral inputs that may influence health behaviors such as regular MNP compliance. In the theoretical literature, the Integrated Behavioral Model (IBM) has been promoted as a model for comprehensively understanding the mechanisms underlying health-related behaviors [16–19]. IBM has been found to be a reliable and valid model for predicting health-related behaviors in underdeveloped communities in LMICs [16–19]. Empirical studies have also found individual behavioral inputs within the IBM model to directly affect health-related behaviors in the short- and long-term. These include knowledge and skills [20], environmental constraints [21], the salience of information [22], and individual habits [21], as well as belief-based constructs such as attitude, peer norms, and personal agency [23]. To date, however, these behavioral inputs have not been studied in relation to MNP adherence in China.

The overall goal of this study was to investigate the behavioral inputs that would influence MNP adherence among caregivers of infants and toddlers in rural western China. First, we described the rates of MNP adherence of caregivers. Second, using IBM, we examined the correlations of specific behavioral inputs to MNP adherence, as well as the relative importance of each input.

## Methods

### Study design and participants

This study uses cross-sectional field survey data collected in 2019 from six rural counties in China’s western Sichuan Province. The population studied was caregivers with infants and toddlers aged 6–24 months. The sample size was decided by the rule suggested by Mitchell that there should be 10 to 20 times as many cases as parameters to estimate in structural equation modeling analysis [24]. An optimal ratio of 20:1 was used to estimate the most conservative sample size for increased confidence in the results [25]. The calculated sample size required was 940. The research team followed a multistage cluster sampling protocol to select the study sample. First, six MNP program counties in Sichuan were randomly selected for inclusion in the study. Next, six sample townships were randomly selected within each sample county. Townships that housed the county seat (which are typically more urbanized) were excluded. Third, within each selected township, the research team randomly selected seven villages with populations above 800. If there were not enough villages with populations above 800 within a township, two neighboring villages were combined and considered one village-level sampling unit. Finally, a list of all households with registered births in the target age range (6–24 months at the time of the survey) was obtained from local health officials in each sample village, and 990 eligible participants (primary caregivers) were identified. The research team excluded 23 participants (2.3%) due to missing data for key variables. The final sample size is therefore 958 infant caregivers from 252 villages in six rural counties.

### Ethical approval

for the current study was granted by the Sichuan University medical ethics committee (Approval # K2018103) prior to data collection. Parents or legal guardians of the children provided written informed consent for their own and their child’s involvement in this study.

### Measures

Quantitative data were collected through structured survey interviews administered by trained enumerators. The survey was developed using the Integrated Behavioral Model (IBM) and adapted for MNP adherence behavior following qualitative interviews with caregivers and MNP staff and two rounds of Delphi expert consultation. The questionnaire was then pre-tested in three non-sample villages and revised for validity, leading to the final version of the questionnaire. Further details about the development of the questionnaire and content validity tests are available in Additional file 1. The survey collected data on MNP adherence, behavioral inputs and demographic covariates.

### MNP adherence

MNP adherence among caregivers was the outcome variable in this study. Based on the recommendations of the WHO and the Chinese MNP program office that children should be fed four to seven sachets of MNP per week from six months till two years old [26, 27], a latent variable for MNP adherence was generated using three self-report indicators: (a) “the number of MNP sachets fed in the past seven days”; (b) “the average number of MNP sachets fed per week”; (c) “the continuity and interruption of MNP feeding from the time the infant was six months of age to the time of the survey.” These three indicators measure adherence to MNP guidelines at the time of the survey, during a typical week and in the long-term.

### Behavioral inputs influencing MNP adherence

**Fig. 1:**
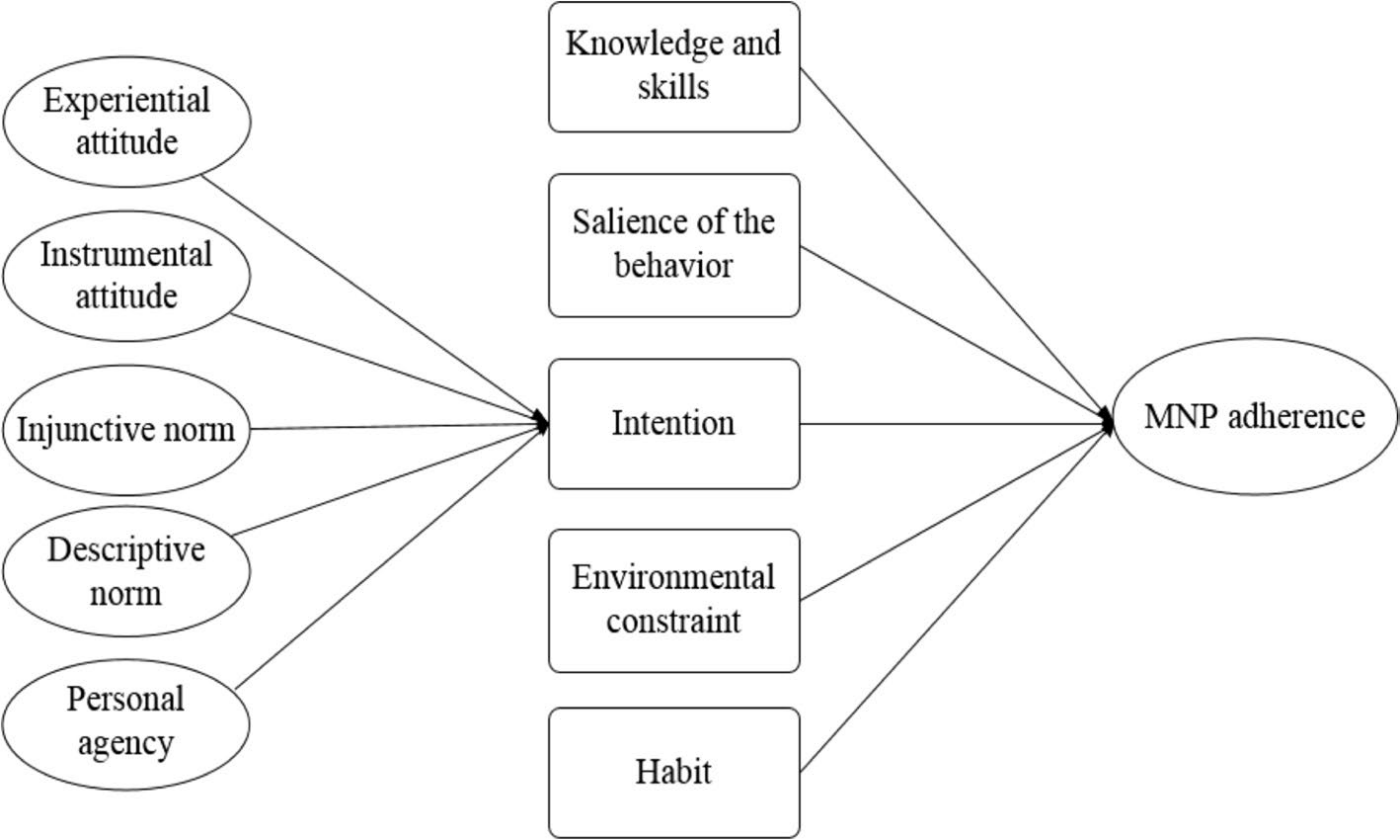
Initial Conceptual Model for the MNP adherence

Figure 1 presents the conceptual model for behavioral inputs influencing MNP adherence. The IBM includes five behavioral inputs that directly influence health-related behaviors: knowledge and skills, intention, salience, environmental constraints, and habits. In this study, knowledge and skills were measured using eight questions about MNP, including sachet ingredients, function, dosage, method of use, storage conditions, abnormal reactions after feeding, whether MNP can replace breast milk, and whether opened MNP sachets can be administered the next day. Correct responses were scored as one point, and incorrect responses were scored as zero points. Responses were summed to create a final caregiver knowledge score. Intention was measured by asking the extent to which the caregiver planned to adhere to MNP feeding guidelines in the future. To measure salience of behavior, which refers to factors that remind people to consistently carry out a certain behavior, caregivers were asked how often their doctor talked to them or reminded them about MNP. To assess environmental constrains, caregivers were asked whether MNP had ever been out of stock in their area. Finally, habits were assessed by asking caregivers whether they had previous experience administering MNP to infants. Questions regarding intention, salience, environmental constraints, and habits were measured on a 5-point Likert scale ranging from 1 = not at all to 5 = completely.

The IBM also includes five belief-based constructs that act as indirect behavioral inputs. These include instrumental attitude, experiential attitude, injunctive norms, descriptive norms, and personal agency. Instrumental attitude refers to the caregiver’s beliefs about the outcomes of adhering to MNP. Experiential attitude refers to the caregiver’s personal feelings about feeding MNP. Injunctive norms (also referred to as subjective norms) include the caregiver’s perceptions of how important individuals in their lives (e.g., family members) feel about MNP, as well as the caregiver’s own motivation to comply with the attitudes and instructions of those individuals. Descriptive norms refer to the caregiver’s perception of MNP feeding behavior among others in their social networks. Finally, personal agency refers to the caregiver’s self-efficacy and perceived control in feeding their child MNP. For all indirect behavioral inputs, latent variables were constructed from responses to multi-item questionnaires. Responses to each item were measured on a 5-point Likert scale ranging from 1 = not at all to 5 = completely. (For the full description of scales and items used to construct the latent variables, see Additional file 1.)

The ellipses represent latent constructs consisting of multiple measurement components. The rectangles represent observation variables. The measurement components for latent constructs have been omitted for clarity.

## Covariates

Several covariates were included as possible confounders. These include primary caregiver age and education, whether the primary caregiver was the parent or grandparent, infant age, and family economic status. To assess family economic status, a household asset index was generated using polychoric principal components analysis based on whether the household owned or had access to a water heater, washing machine, refrigerator, air conditioner, television, computer, motorcycle, and car or truck.

### Statistical analysis

To identify behavioral inputs that may influence MNP adherence behavior, data were analyzed using structural equation modeling (SEM) with demographic characteristics included as covariates. Confirmatory factor analysis was used to test the reliability and validity of the latent variables. Composite reliability (CR) and construct validity of the latent factors were assessed at the measurement level. The composite reliability is supported when CR exceeds 0.70. The convergent validity is supported when the average variance extracted (AVE) exceeds 0.50. A Pearson’s correlation coefficient was estimated for each latent variable. The discriminant validities of the constructs are supported when the square-root of the AVE for each latent variable exceeds its correlation with other latent variables. The goodness-of-fit of the SEM model was tested using conventional fit indices, including the adjusted goodness-of-fit index (AGFI), comparative fit index (CFI), Tucker-Lewis index (TLI), root-mean-square error of approximation (RMSEA), and standardized root mean square residual (SRMR) [28]. Values exceeding 0.90 for the AGFI, CFI, and TLI and values below 0.08 for the RMSEA and SRMR, were considered indicative of ideal model fit. Given the non-normality of five-point response scale, robust corrections were applied using the Bollen-Stine bootstrap procedure to estimate corrected Chi-square values [29, 30].

The standardized direct effects, indirect effects, and total effects of the SEM analysis were estimated using maximum likelihood estimation (MLE). Non-multivariate normal data would cause biased estimates of standard errors but unbiased parameter estimates [31]. To correct the standard errors and adjust robustness of MLE to multivariate nonnormality, we used 2000 bootstrap samples with 95% bias-corrected confidence intervals to determine the significance of results, the mediation effect and subsequently validated the proposed model [29, 32]. The path coefficient reflects the response of the dependent variable to a unit change in an explanatory variable while the other variables in the model are kept constant [33]. We estimated the effect sizes of all the significant factors by calculating Cohen’s *f* ^2^ statistic. Cohen’s *f* ^2^ effect sizes lower than 0.02 were considered very small; between 0.02 and 0.15 are considered small; between 0.15 and 0.35 are considered moderate; and effect sizes larger than 0.35 are considered large [34]. In addition to analyzing the full sample, multi-group analysis was performed by type of caregiver (parents and grandparents). A post hoc power analysis was also conducted based on the observed effect size, an α = 0.05, and the smallest sample size used in the SEM analyses.

Statistical analyses were performed using Stata 15.0 (StataCorp LLC) and AMOS 22.0 (IBM Corp., Armonk, NY, USA). Post hoc power analysis was conducted using G*Power 3.1.9 software to validate sample size and confirm sufficient statistical power (> 0.8). Using RGui 3.2.2 to apply the RMSEA to calculate the test performance (power) of the SEM model. *P*-values of 0.05 and below were considered statistically significant. The results of the reliability and validity analysis and post hoc power analysis are reported in Additional file 1.

## Results

### Participant characteristics

The demographic characteristics of study participants are reported in Table 1. The sample included 958 primary caregivers of infants and toddlers aged 6 to 24 months. About 70.56% of caregivers were parents, with a mean caregiver age of 36.13 years (SD = 13.09). Most caregivers (88.06%) had a junior high school education level or below.

**Table 1:** Socio-demographic characteristics of the respondents

Characteristics (*N* = 958)	Percent or Mean (as appropriate)
Infant and toddler age(months)	
6–12	25.37
13–18	35.18
19–24	39.46
Ethnicity	
Han	33.19
Tibet	32.26
Yi	34.55
Generation of primary caregiver	
Parents	70.56
Grandparents	29.44
Caregiver age(years) (± SD)	36.13 ± 13.09
Caregiver education	
9 years and below	88.06
Above 9 years	11.94
Household assets	
Relatively lower	50.10
Relatively higher	49.90

### MNP adherence

The MNP adherence indicators of caregivers are presented in Table 2. The weighted average across all caregivers shows 53.02% had fed infants and toddlers four or more MNP sachets in the previous seven days, and 63.67% of caregivers regularly administered four or more bags of MNP to infants and toddlers from six months of age to the time of the survey. Only 22.86% of all caregivers consistently fed their infants and toddlers MNP without interruption from the start of MNP distribution at six months of age to the time of the survey. Compared to parent caregivers, grandparents had a higher degree of MNP adherence on all three behavioral indicators (*P* < 0.001).

**Table 2:** MNP adherence behavior of full sample and among parent and grandparent caregiver subgroups

	Total (*N* = 958)		Parents (*n* = 676)		Grandparents (*n* = 282)		*P* from test of difference
	*n* (%)		*n* (%)		*n* (%)		
**Number of MNP sachets fed in the past 7 days** ^a^							< 0.001
0 bag	467(48.75)		351 (51.92)		116 (41.13)		
1–3 bags	78(8.14)		60 (8.88)		18 (6.38)		
4–6 bags	51(5.32)		36 (5.33)		15 (5.32)		
7 bags	362(37.79)		229 (33.88)		133 (47.16)		
**Average number of MNP sachets fed per week** ^b^							< 0.001
0 bag	254 (26.51)		190 (28.11)		64 (22.70)		
1–3 bags	94 (9.81)		75 (11.09)		19(6.74)		
4–6 bags	53 (5.53)		40 (5.92)		13(4.61)		
7 bags	557 (58.14)		371 (54.88)		186(65.96)		
**Continuity of feeding behavior** ^c^							< 0.001
Never feeding	179(18.68)		128(18.93)		51(18.09)		
Have fed before	145(15.14)		114(16.86)		31(10.99)		
Irregular feeding	160(16.70)		125(18.49)		35(12.41)		
Occasionally interrupted feeding	255(26.62)		170(25.15)		85(30.14)		
Consistent feeding	219(22.86)		139(20.56)		80(28.37)		

^a^Number of MNP sachets fed in the past seven days was used to assess current caregiver adherence. ^b^Average number of MNP sachets fed per week was used to assess typical MNP adherence. Four bags and above correspond to MNP adherence specified by WHO and the Chinese MNP program office. ^c^Continuity of feeding behavior was used to assess long-term MNP adherence by measuring caregiver MNP feeding behaviors from the time the infant was six months of age to the time of the survey.

### Reliability and validity analyses

The composite reliability (CR) of measurement items ranged from 0.702 to 0.887, indicating high composite reliability of the measurement items in all the latent constructs. The average variance extracted (AVE) fell within the acceptable range, which offered evidence of convergent validity [35, 36]. All the square roots of AVE were greater than the correlation involving the constructs, confirming the discriminant validity of the constructs. The correlation matrix, reliability, and validity of measuring models are shown in Table S2 in Additional file 1.

### Goodness-of-fit analysis

An evaluation of the overall goodness-of-fit of the SEM model was conducted to determine its suitability for analyzing the impact of IBM constructs on MNP adherence. Nonparametric Bollen–Stine bootstrapping estimation was used to improve the robustness of the SEM analysis and address potential issues from nonlinearity and multivariate non-normality (Multivariate kurtosis = 25.299, c.r. = 10.12). Most of the fitting indices were within, or close to, the reasonable range suggested by Bentler [28], indicating adequate fit (Additional file 1: Table S3).

### Behavioral inputs and MNP adherence

#### Results of structural equation modeling for IBM constructs

**Fig. 2:**
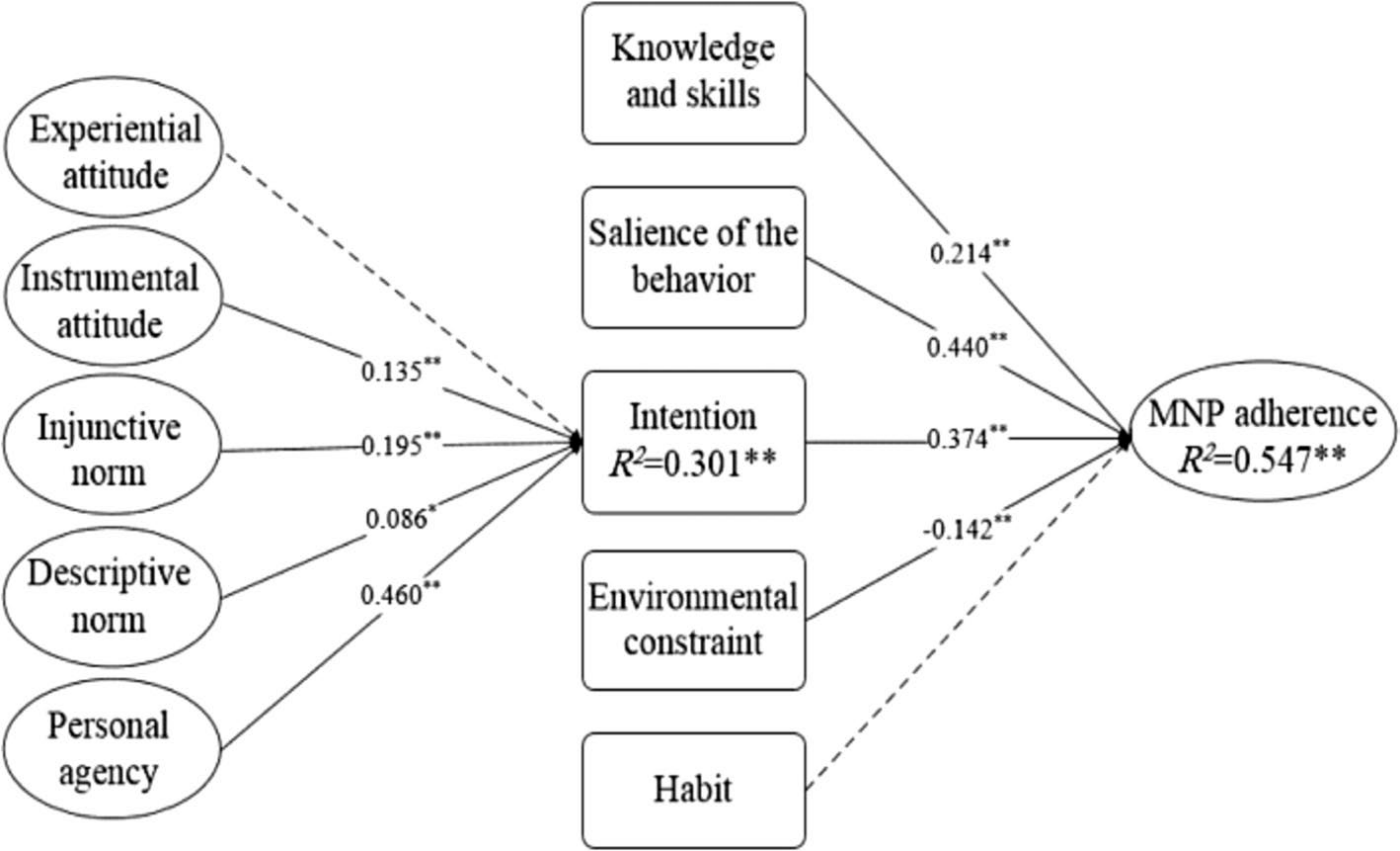
Adjusted SEM based on IBM framework for the MNP adherence

Figure 2 shows the results of the SEM analysis. Three of the five direct behavioral inputs had significant positive effects on MNP adherence, including knowledge and skills (β = 0.214, *P* < 0.001), the salience of behavior (β = 0.440, *P* < 0.001), and intention (β = 0.374, *P* < 0.001). Environmental constraints showed a significant negative effect on MNP adherence behavior (β=-0.142, *P* < 0.001). Additionally, four of the five indirect behavioral inputs predicted MNP adherence through intention, including instrumental attitude (β = 0.135, *P* < 0.001), injunctive norms (β = 0.195, *P* < 0.001), descriptive norms (β = 0.086, *P* < 0.050), and personal agency (β = 0.460, *P* < 0.001). Habit showed no significant effect on behavior, and experiential attitude did not significantly predict MNP adherence through intention.

The ellipses represent latent constructs consisting of multiple measurement components. Path estimates with solid lines were hypothesized to be significant, broken lines were hypothesized to be non-significant. The measurement components of the latent constructs and effects of control variables that were statistically significant (infants and toddlers age, caregiver generation, and family economic status) have been omitted for clarity. Detail results of the model are shown in Fig S1 in Additional file 1. * *P* < 0.05 ** *P* < 0.001.

Parameter estimates for effects among constructs in the SEM model are presented in Table 3. The results revealed statistically significant medium-sized effects of salience and intention, and small-sized effects of knowledge and skills, personal agency, environmental constraints, and caregiver generation on MNP adherence. By comparison, the effects of the injunctive norms, instrumental attitude, and descriptive norms on MNP adherence were very small, though the effects remain statistically significant. Among all behavioral inputs, the salience of behavior had the largest total effect on MNP adherence (total effect = 0.440, *P* < 0.001, Cohen’s *f* ^2^ = 0.227), followed by behavioral intention (total effect = 0.374, *P* < 0.001, Cohen’s f^2^ = 0.156) and knowledge and skills (total effect = 0.214, *P* < 0.001, Cohen’s *f* ^2^ = 0.058). Among the indirect variables mediated by intention, the personal agency showed the greatest effect on MNP adherence (total effect = 0.172, *P* < 0.001, Cohen’s *f* ^2^ = 0.033).

**Table 3:** Parameter estimation of structural equation modeling for MNP adherence

Ranking^a^	Behavioral Input	Unstd. β^b^	S.E.	Z	Std. β^c^	Total effects^d^(CI_95_)	Cohen’s *f* ^2 e^
1	Salience	0.377^**^	0.026	14.416	0.440	0.440(0.372, 0.501)	0.227
2	Intention	0.342^**^	0.027	12.581	0.374	0.374(0.308, 0.433)	0.156
3	Knowledge and skills	0.138^**^	0.019	6.428	0.214	0.214(0.120, 0.264)	0.058
4	Personal agency	0.902^**^	0.086	1.448	0.460	0.172(0.119, 0.218)	0.033
5	Environmental constraints	-0.476^**^	0.096	-4.947	-0.142	-0.142(-0.194, -0.090)	0.030
6	Caregiver generation	0.282^*^	0.068	4.159	0.119	0.119(0.052, 0.165)	0.029
7	Infant and toddler age	0.019^*^	0.007	2.839	0.081	0.081(0.022, 0.143)	0.011
8	Injunctive norm	0.413^**^	0.074	5.547	0.195	0.073(0.043, 0.105)	0.010
9	Family economic status	-0.063^*^	0.028	-2.285	-0.065	-0.065(-0.146, -0.022)	0.005
10	Instrumental attitude	0.069^**^	0.015	4.432	0.135	0.050(0.022, 0.082)	0.003
11	Descriptive norm	0.106^*^	0.040	2.671	0.086	0.032(0.007, 0.060)	0.001

^a^The ranking is based on the total effect of each factor on MNP adherence behavior in descending order of magnitude. ^b^Unstd.β: Unstandardized path coefficient. ^c^Std. β: Unstandardized path coefficient. ^d^The total effect of the behavioral input on the MNP adherence. For indirect factors (i.e., personal agency, injunctive norm, instrumental attitude, descriptive norm), the total effect is mediated by the intention. ^e^Cohen’s *f* ^2^ < 0.02, Cohen’s *f*^2^ ≥ 0.02, Cohen’s *f*^2^ ≥ 0.15, and Cohen’s *f*^2^ ≥ 0.35 represent very small, small, medium, and large effect sizes, respectively. *P < 0.05 **P < 0.001.

### Multi-group analysis by type of caregiver

Table 4 presents the results for the multi-group analysis of predictors of MNP adherence among parent and grandparent caregivers. Although grandparents had a higher degree of MNP adherence on average (Table 2), the results in Table 4 indicate that behavioral inputs were similar for both groups of caregivers. The constrained and unconstrained models did not significantly differ in structural weights (P = 0.379). Thus, the constructs of the model were reasonably invariant and comparable across parent and grandparent groups.

**Table 4:** Parameter estimation of SEM for MNP adherence for parent and grandparent caregivers

Behavioral Input	Parents (*n* = 676)			Grandparents (*n* = 282)	
	Std.β^a^	Total effect^b^(CI_95_)		Std.β^a^	Total effect^b^(CI_95_)
Salience	0.452^**^	0.452(0.374, 0.529)		0.379^**^	0.379(0.258, 0.500)
Intention	0.371^**^	0.371(0.300, 0.442)		0.374^**^	0.374(0.242, 0.495)
Knowledge and skills	0.226^**^	0.226(0.137, 0.312)		0.212^**^	0.212(0.095, 0.335)
Personal agency	0.492^**^	0.182 (0.126, 0.243)		0.312^**^	0.117 (0.043, 0.215)
Environmental constraints	-0.137^**^	-0.137(-0.206, -0.08)		-0.174^**^	-0.174(-0.27, -0.077)
Infants and toddlers age	0.085^*^	0.085(0.016, 0.156)		0.004	0.004(-0.100, 0.109)
Injunctive norm	0.192^**^	0.071 (0.04, 0.113)		0.155^*^	0.058(0.005, 0.128)
Family economic status	-0.082^*^	-0.082(-0.181, 0.019)		-0.032	-0.032(-0.162, 0.104)
Instrumental attitude	0.112^**^	0.042(0.009, 0.079)		0.196^**^	0.073(0.022, 0.155)
Descriptive norm	0.069	0.025(-0.004, 0.057)		0.130^*^	0.049(0.004, 0.098)

^a^Std. β: Standardized path coefficient. ^b^The total effect of the behavioral input on the MNP adherence. For indirect factors(i.e., personal agency, injunctive norm, instrumental attitude, descriptive norm), the total effect is mediated by the intention.

* *P* < 0.05 ** *P* < 0.001.

## Discussion

This study examined caregiver adherence to MNP feeding and identified behavioral determinants of MNP feeding adherence among caregivers of young children in rural western China. To our knowledge, this is the first study to examine behavioral inputs in MNP adherence using the IBM framework. The SEM method allows us to examine the complex relations of multiple direct and indirect behavioral inputs on the MNP adherence behaviors of caregivers, which can inform strategies to increase MNP adherence.

### MNP adherence

Overall, the study found MNP adherence rates to be relatively low. Slightly more than half of all caregivers had fed infants and toddlers four or more MNP sachets in the previous seven days, which is similar to the rates of 49-64% in other studies conducted in rural western China [11, 13–15].Less than a quarter of all caregivers consistently fed their infants and toddlers MNP without interruption from the start of MNP distribution at six months of age to the time of the survey, 15.14% of caregivers had fed their children MNP but were no longer currently feeding, which is similar to a study in Yunnan Province where the MNP premature discontinuation rate was 12.91% [37]. Overall, MNP adherence indicators in this study were all below the 70% desired adherence rate for MNP programs [38], and it also needs to be improved compared to the 87% of caregivers who gave children one sachet of MNP per day in a study conducted in Africa [39].

### Behavioral inputs in MNP adherence

Most of the behavioral inputs examined predicted MNP adherence directly or indirectly, with salience and intention showing the strongest relation to MNP adherence. Salience, represented by doctor-communicated reminders to caregivers to feed infants and toddlers the MNP sachets, had the greatest effect on MNP adherence with a medium effect size (Cohen’s *f* ^2^ = 0.227). This finding is similar to studies conducted in other LMICs, which have found that increased communication with health providers regarding MNP and nutritional supplication increases MNP adherence among caregivers [40–43]. In China, rural caregivers tend to have less access to health information and lower levels of education, and community doctors responsible for distributing MNP are the primary source of MNP-related information [44]. Increased communication between health providers and rural households, either through incentives for providers to increase communication or through the employment of additional health providers such as community health workers, may increase salience and raise MNP adherence. Food supplementation programs in similar contexts have found success through meticulous planning focused towards improving health worker motivation, enhanced communication, and involvement of multiple stakeholders [45].

Intention also had relatively higher and significant effects on MNP adherence among caregivers with a medium effect size (Cohen’s *f* ^2^ = 0.156). This finding is in line with the propositions of IBM, and with previous research in rural China [15]. Importantly, intention is also a mediating factor for indirect behavioral inputs on MNP adherence, namely personal agency, as well as attitudes, and norms. Personal agency is particularly relevant to individuals facing obstacles to specific actions [46] and can be affected by the results of past behavior, the demonstration effect of positive or negative experiences of others, evaluation and persuasion of others, and awareness of the weakening of the obstacles [47]. In the context of this study, it could be that caregivers face difficulties in feeding MNP (such as the child refusing to eat the MNP), which may reduce intention to feed MNP by diminishing caregivers’ self-efficacy in MNP feeding. Strategies to help caregivers overcome obstacles in MNP feeding, such as coaching them on effective tactics to feed their children MNP, may improve the personal agency of caregivers, thus promoting both intention to feed children MNP and overall MNP adherence.

These findings suggest possible barriers to MNP adherence among “inclined abstainers”, that is, people who may be willing to feed their children MNP but are unable to adhere to MNP feeding for various reasons [48, 49]. Multiple studies have indicated that many individuals report positive intentions toward health behaviors but fail to act on these intentions [50, 51]. In this study, policies and programs aimed at increasing salience around MNP among caregivers, as well as increasing caregivers’ feelings of personal agency in MNP feeding, may promote increased adherence among a substantial proportion of inclined abstainers.

### MNP adherence among parents and grandparents

Finally, we found a higher degree of MNP adherence among grandparent caregivers compared to parent caregivers. This result is consistent with another study in rural China that have found generational differences in MNP adherence [52]. In our fieldwork, we learned that many young parents who are not familiar with MNP have a distrust of “free products that have no guarantee of quality,“ which may explain the reduced MNP adherence among parents. To increase MNP adherence among parent caregivers, policymakers should increase the visibility of MNP to gain the trust of young caregivers. One way to do so is to increase communication by health providers on the importance of MNP; this may also increase salience and personal agency regarding MNP feeding, as discussed above. Interestingly, previous research has found that older caregivers (e.g., grandparents) are slower to adopt informed practices, potentially contributing to the lag in their behavioral changes and poorer feeding practices than do younger caregivers [51]. The increased adherence to MNP among grandparents suggests that MNP may help to compensate for poorer feeding practices among grandparent caregivers and prevent developmental delays in children under their care.

This study makes three key contributions to the literature. First, whereas previous studies have used a single behavioral indicator of MNP adherence, this study employs three indicators (current adherence, average adherence, and long-term adherence) to provide a more comprehensive picture of MNP adherence behavior among caregivers. Second, this is the first study to apply the Integrated Behavioral Model (IBM) towards identifying direct and indirect inputs in MNP adherence. The application of IBM has led to a higher degree of explanatory power for behavior compared to other behavioral theories; in previous studies and meta-analyses, the amount of variance explained in behavior was 20–30% [53–55]. Using IBM, this study studies and identifies the factors that directly and indirectly influence behavior accounting for a total of 54.7% of the variance in MNP adherence among the sample. The findings also help to identify potential mechanism through which policy makers and healthcare practitioners may effectively increase MNP adherence.

This study has the following limitations. First, this study is cross-sectional design. Although model-fit indices indicated consistency between the causal assumptions of our SEM model and our data, the causal direction of the proposed effects was inferred from IBM theory alone. In addition, among the 32 MNP project counties, only six counties were sampled for the survey. To compensate for this limitation, we stratified the sample counties according to the ethnic composition ratio of the MNP project counties (1:2 of Han to ethnic minorities) and randomly selected six counties scattered across Sichuan province, with different economic levels and cultural backgrounds, as well as different landscapes of plains, highlands, and mountains. Nevertheless, the results may not be generalizable to other regions or contexts. Future studies using experimental designs are needed to establish causal relations among the model variables and determine whether interventions targeting the identified behavioral inputs effectively increase MNP adherence among rural caregivers.

## Conclusion

The current study established a hypothesized model on MNP adherence of infants and toddlers caregivers in rural China. The results suggest that the additional effort is needed to improve MNP adherence. Our empirical analysis confirmed key factors with small to medium effect sizes (e.g., salience, intention, and personal agency) that may offer some potential in promoting MNP adherence behavior. This study provided the necessary information to design behavior change strategies that might be helpful for the improvement of micronutrient home fortification programs and enhance the effectiveness of these programs. In sum, then, we believe that researchers and policymakers working in middle-income economies can learn from our findings and may be interested in our conclusions.

## Electronic supplementary material

**Additional file 1: Table S1.** Full set of latent variables domains and itemsbased on Integrated Behavioral Model framework. **Table S2.** Confirmatory factor analysis for latent variables. **Table S3.** Evaluation of the goodness-of-fit of the structural equation model. **Fig. S1.** Detail results of the Adjusted SEM based on IBMframework for the MNP adherence. 

**Additional file 2.**

## Data Availability

All data generated during this study are included in the supplementary information files.
