# Supplementary material for: Adherence to micronutrient powder for home fortification of foods among infants and toddlers in rural China: a structural equation modeling approach
Source: BMC Public Health. 2022 Dec 2;22:2250. doi: 10.1186/s12889-022-14731-3 (PMC9719183; doi:10.1186/s12889-022-14731-3)
Supplement: Supplementary file 1 — Additional file 1: Table S1. Full set of latent variables domains and itemsbased on Integrated Behavioral Model framework. Table S2. Confirmatory factor analysis for latent variables. Table S3. Evaluation of the goodness-of-fit of the structural equation model. Fig. S1. Detail results of the Adjusted SEM based on IBMframework for the MNP adherence. [file 12889_2022_14731_MOESM1_ESM.docx]

**Details about the development of the questionnaire and content validity tests:**

We conducted a systematic literature search using databases such as PubMed, Web of Science, Embase, CAB Abstracts & MEDLINE (OVID), Cochrane Library, China National Knowledge Infrastructure (CNKI), Wanfang, and VIP. Our search terms included items related to those belief-based measures. In addition, we conducted qualitative interviews with parents and MNP staff in our previous research to learn in advance about the experience of distributing, receiving, and feeding MNP in the field. After developing the survey questionnaire, 12 experts with backgrounds in maternal and child health, health behavior, nutrition, epidemiology and health statistics, social science, and psychology were invited consult on the importance, operability, and sensitivity of each item.

The questionnaire was then pre-tested in one Han and two minority non-sample villages. We analyzed the reliability and validity of the survey, with Cronbach`s coefficients ranging from 0.702-0.826 and KMOs ranging from 0.662-0.762 for each dimension. We also refined the field operation procedures to accommodate local contexts, including listing local names for YYB, listing comprehensive and differentiated items of knowledge and attitudes towards MNP, and implementing the locally used calendar in minority settings to determine each infant’s age with a high degree of accuracy.

**Table S1** Full set of latent variables domains and items based on Integrated Behavioral Model framework

| Domain | Items | Answers |
| --- | --- | --- |
| instrumental attitude | a. YYB^a^ can prevent malnutrition in babies | (1)‘strongly disagree’ to (5) ‘strongly agree’ |
|  | b. YYB can prevent anemia in babies |  |
|  | c. YYB can make babies grow taller or stronger |  |
| experiential attitude | a. It is convenient to get YYB from doctors |  |
|  | b. The process of preparing YYB for baby is simple |  |
|  | c. YYB knowledge is easy for me to understand |  |
| Injunctive norms^a^ (normative belief about important referent individuals) |  | (1)‘not at all’ to (5)‘completely’ |
|  | Whether the following referent individuals approve of YYB feeding |  |
|  | a. Infant’s and toddler’s parents |  |
|  | b. Infant’s and toddler’s maternal grandparents |  |
|  | c. Infant’s and toddler’s paternal grandparents |  |
|  | d. Caregiver’s friends |  |
|  |  |  |
|  | e. Village cadres |  |
| Injunctive norms^a^ (motivation to comply with that referent) | The extent to which caregivers will comply with advice from the following referent individuals |  |
|  | a. Infant’s and toddler’s parents |  |
|  | b. Infant’s and toddler’s maternal grandparents |  |
|  | c. Infant’s and toddler’s paternal grandparents |  |
|  | d. Caregiver’s friends |  |
|  | e. Doctors |  |
|  |  |  |
| Descriptive norms | a. Have you ever seen or heard of other people's toddler eating YYB? | (1)‘never’ to (5) ‘always’ |
|  | b. Have you ever seen or heard of other people's toddler like to eat YYB? |  |
|  | c. Have you ever seen or heard of other people's toddler have a positive change after taking YYB? |  |
| Personal agency | a. Are you confident that you can deal efficiently with difficulties in feeding YYB? | (1)‘not at all’ to (5)‘completely’ |
|  | b. Are you confident that you can keep feeding YYB until your baby is two years old? |  |
|  | c. Is it under your control to insist on feeding YYB? |  |

^a^ Injunctive norms was computed by multiplying the caregiver normative belief by motivation to comply with the referent.

**Table S2** Confirmatory factor analysis for latent variables

| Domains | CR | AVE | 1 | 2 | 3 | 4 | 5 | 6 |
| --- | --- | --- | --- | --- | --- | --- | --- | --- |
| 1. Adherence behavior | 0.887 | 0.725 | 0.85^a^ |  |  |  |  |  |
| 2. Experiential attitude | 0.702 | 0.445 | 0.38^**b^ | 0.67^a^ |  |  |  |  |
| 3.Instrumental attitude | 0.826 | 0.651 | 0.38^**b^ | 0.36^**b^ | 0.81^a^ |  |  |  |
| 4. Injunctive norm | 0.799 | 0.431 | 0.27^*b^ | 0.32^*b^ | 0.43^**b^ | 0.66^a^ |  |  |
| 5. Descriptive norm | 0.841 | 0.650 | 0.24^*b^ | 0.27^*b^ | 0.30^*b^ | 0.30^*b^ | 0.81^a^ |  |
| 6. Personal agency | 0.724 | 0.428 | 0.56^**b^ | 0.63^**b^ | 0.59^**b^ | 0.42^**b^ | 0.30^*b^ | 0.65^a^ |

^a^ Table diagonally c is the square root of AVE. ^b^ The Pearson's correlation coefficient *r_s_* for each latent variable. The discriminant validities of the constructs are supported when the square-root of the AVE for each latent variable exceeds its correlation with other latent variables.

* *P* < 0.05 ** *P* < 0.001

**Table S3** Evaluation of the goodness-of-fit of the structural equation model

| Indicators | Measurement standard | Fitted value |
| --- | --- | --- |
| Bollen-Stine $^{2}$ ^a^ | - | 213.353 |
| NC($^{2}$/df) | 1~3 | 1.036 |
| GFI (goodness of fit index) | ＞0.9 | 0.967 |
| AGFI (adjusted goodness of fit index) | ＞0.9 | 0.957 |
| RMSEA (root mean square error of approximation) | ＜0.08 | 0.006 |
| SRMR(Standardized Root Mean Square Residual) | ＜0.08 | 0.146 |
| TLI(Tucker-Lewis index) | ＞0.9 | 0.999 |
| CFI (comparative fit index) | ＞0.9 | 0.999 |
| IFI (incremental fit index) | ＞0.9 | 0.999 |
| power | ＞0.80 | 0.999 |

^a^ Bollen-Stine bootstrap $^{2}$ 213.353 was a mean value of 2000 times while the $^{2}$value estimated by the pre-correction maximum likelihood method was 2003.759 (p < 0.001)


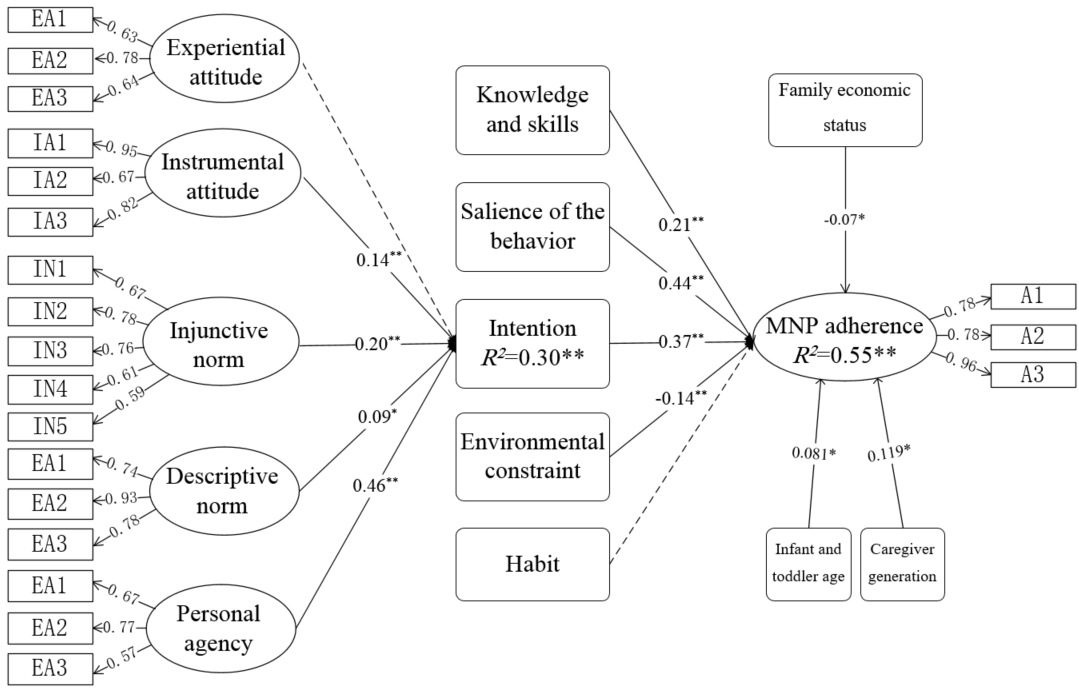


**Fig. S1.** Detail results of the Adjusted SEM based on IBM framework for the MNP adherence.

The ellipses represent latent constructs consisting of multiple measurement components. Path estimates with solid lines were hypothesized to be signiﬁcant, broken lines were hypothesized to be non-signiﬁcant.

* *P* < 0.05 ** *P* < 0.001.

**Result of post hoc power analysis**

Post hoc power analysis was conducted based on the observed effect size (*f*^2^ estimate of 0.2), an α = 0.05, and the smallest sample size in the above SEM analysis (n=282 in the multi-group analysis) to represent a conservative estimate of power. A post hoc power of 0.99 was achieved, demonstrating that adequate power to detect a moderate effect in this sample.
